# HPV Infection Affects Human Sperm Functionality by Inhibition of Aquaporin-8

**DOI:** 10.3390/cells9051241

**Published:** 2020-05-17

**Authors:** Giorgia Pellavio, Federica Todaro, Paola Alberizzi, Claudia Scotti, Giulia Gastaldi, Marco Lolicato, Claudia Omes, Laura Caliogna, Rossella E. Nappi, Umberto Laforenza

**Affiliations:** 1 Department of Molecular Medicine, Human Physiology Unit, University of Pavia, I-27100 Pavia, Italy; giorgia.pellavio@gmail.com (G.P.); gastaldi@unipv.it (G.G.); 2 Center for Reproductive Medicine, Obstetrics and Gynecology Unit, Fondazione IRCCS Policlinico San Matteo, University of Pavia, I-27100 Pavia, Italy; todarofederica@gmail.com (F.T.); R.Nappi@smatteo.pv.it (R.E.N.); 3 IRCCS-Fondazione Policlinico San Matteo, Pavia I-27100, Italy; p.alberizzi@smatteo.pv.it (P.A.); L.Caliogna@smatteo.pv.it (L.C.); 4 Department of Molecular Medicine, Unit of Immunology and General Pathology, University of Pavia, I-27100 Pavia, Italy; claudia.scotti@unipv.it (C.S.); marcogaetano.lolicato@unipv.it (M.L.); 5 Center for Health Technologies (CHT), University of Pavia, I-27100 Pavia, Italy; 6 Center for Reproductive Medicine, Obstetrics and Gynecology Unit, Fondazione IRCCS Policlinico San Matteo, I-27100 Pavia, Italy; claudia.omes@unipv.it

**Keywords:** water channel, oxidative stress, sterility, sperm motility, aquaporin

## Abstract

Human sperm cells express different aquaporins (AQPs), AQP3, 7, 8, 11, which are localized both in the plasma membrane and in intracellular structures. Besides cell volume regulation and end stage of cytoplasm removal during sperm maturation, the role of AQPs extends also to reactive oxygen species (ROS) elimination. Moreover, oxidative stress has been shown to inhibit AQP-mediated H_2_O_2_ permeability. A decrease in AQPs functionality is related to a decrease in sperm cells number and motility. Here we investigate the possible effect of human Papillomavirus (HPV) on both expression and function of AQPs in human sperm cells of patients undergoing infertility couple evaluation. Stopped-flow light-scattering experiments demonstrated that HPV infection heavily reduced water permeability of sperm cells in normospermic samples. Confocal immunofluorescence experiments showed a colocalization of HPV L1 protein with AQP8 (Pearson’s correlation coefficient of 0.61), confirmed by co-immunoprecipitation experiments. No interaction of HPV with AQP3 and AQP7 was observed. A 3D model simulation of L1 protein and AQP8 interaction was also performed. Present findings may suggest that HPV infection directly inhibits AQP8 functionality and probably makes sperm cells more sensitive to oxidative stress.

## 1. Introduction

Persistent infection by High-Risk Human Papillomavirus (HR-HPV) is considered the main cause for human cervix, ano-genital, head, and neck cancer development [1]. Inflammation is one of the tissue responses to HR-HPV infection to promote viral elimination [2]. The resulting oxidative state causes lipid, protein or nucleic acid oxidation which, in turn, can lead to apoptosis, necrosis or cancer development [3]. HR-HPV and oxidative stress are strictly associated for two reasons: (1) early expressed viral proteins E1, E2, E4–E7 are involved in the modulation of cells oxidative state, mainly by increasing reactive oxygen species (ROS) concentration, and (2) late expressed viral proteins L1 and L2 need an oxidative environment for capsomere assembly and to complete HR-HPV life cycle (for a complete review see [4]). 

It is well known that HPV is responsible for inducing cancer, but growing evidence suggests that it also plays a role in causing transient male sub-fertility. As stated by Depuydt and coworkers, these are two sides of the same coin [5]. Even though the occurrence of HPV-associated cancer in man is several-fold (about 15.6) lower than in women [6], there is a growing interest about HPV infection in males because HPV infection was suggested to be detrimental for some sperm parameters [5,7]. Several studies, included in reviews and meta-analysis of the literature, sometimes suggested that HPV infection may represent a risk factor for male infertility, but results are conflicting [5,8,9,10,11]. Lai et al. found a higher incidence of asthenozoospermia in HPV-infected subjects than in those without HPV infection [7]. HR-HPV infection was associated with reduced curvilinear velocity, straight-line velocity, and mean amplitude of lateral head displacement. A decreased progressive motility was also demonstrated by incubating washed sperm with HPV type 6b/11, 16, 18, 31, and 33 DNA fragments, whereas a reduced lateral head displacement was observed only in sperm exposed to HPV type 16 and 6/11 DNA fragments [12]. Progressive motility impairment in sperm of HPV-infected subjects was later confirmed in other studies [13,14]. As a whole, HPV infection was found to decrease sperm progressive motility [12,13,14,15], normal morphology [14,15] and lateral head displacement [7,12] and to increase sperm DNA fragmentation [12,16]. High percentages of antisperm antibodies (ASAs), about 40%, were observed in sperm of HPV-infected subjects [17].

Other studies were unable to demonstrate that HPV infection is associated with poor sperm parameters. Brossfield et al. showed an increased velocity and progression of sperm exposed to L1 HPV-DNA fragments compared with controls [18]. Rintala et al. and Schillaci et al., analyzing sperm parameters both from donors and from males undergoing assisted reproduction, showed that semen volume, sperm concentration, motility and viability were unmodified in HPV-infected subjects [19,20].

The mechanism underlying the possible damage of sperm functionality following HPV infection are still poorly understood. HR-HPV proteins modulate ROS concentration in host cells switching from a eustress to a distress condition [4]. Oxidative stress derives from an imbalance between oxidant and antioxidant systems. The H_2_O_2_ efflux through plasma membranes to the extracellular fluid has been considered an important ROS scavenging system [21,22]. Some aquaporins (AQPs), water channels involved in the transport of water and small molecules, have been recognized to allow the transmembrane diffusion of H_2_O_2_, and for this reason were named peroxiporins: AQP3, AQP5, AQP8 and AQP9 [22,23,24,25,26,27,28,29].

Sperm cells express two types of peroxiporins: AQP3, AQP8. The former is localized in the sperm tail membrane and in a few granules in the head and in the midpiece; the latter is found in the midpiece of spermatozoa, seemingly in the mitochondria and/or in the plasma membrane in the proximity of the midpiece [30,31]. Sperms also express AQP7, localized in the sperm head plasma membrane, and AQP11, confined to the tail and less occasionally in the intracellular structures of spermatozoa [30,32]. Functionally, AQPs were found to be involved in sperm volume regulation and ROS elimination. Sperm number and motility were related with AQP functioning, although a close association with a single AQP paralog could not be demonstrated [30].

This study aims to elucidate whether HPV infection may damage sperm through AQP impairment. To this end, in human ejaculated sperm cells from normospermic and sub-fertile subjects with and without documented HPV-infections we determined: (1) water permeability features measured by a stopped-flow light-scattering method; (2) the interaction between AQPs and HPV capsid protein L1 by confocal fluorescence microscopy (3) the co-immunoprecipitation of HPV with AQP8; (4) a potential L1-AQP interaction mode by protein modelling and docking simulations.

The results reported here provide evidence that HPV binds to AQP8 and inhibits sperm functions.

## 2. Materials and Methods

### 2.1. Sperm Samples

Ninety-four male partners were recruited from infertile couples undergoing infertility evaluation at the Center for Reproductive Medicine, Fondazione IRCCS Policlinico San Matteo (University of Pavia, Pavia, Italy) after informed consent. The consent form was specifically designed for processing of the excess of semen volume collected for diagnostic analysis, in accordance with the Declaration of Helsinki, and with our local Ethical Committee guidelines on the use of residual biological material for research purposes.

Each male produced the semen by masturbation after an abstinence of 2–7 days and semen samples were collected in a sterile plastic container confirmed to be non-toxic for spermatozoa. A routine semen analysis was performed within 1 h of collection, according to the methods described by the World Health Organization (WHO) [33]. The system used for grading motility distinguishes spermatozoa with progressive (PR) or non-progressive (NP) motility from those that are immotile, as reported by the WHO manual (parameters for normospermic patients: *PR* + *NP* ≥ 40%; *PR* ≥ 32%) [33].

Samples were divided into two groups on the basis of their characteristics: *group* 1–67 were from subjects defined *normospermic* based on the following parameters: number of spermatozoa ≥ 15 × 10^6^/mL, progressive spermatozoa ≥ 4.8 mil × 10^6^/mL and physiological viability ≥ 58%); *group 2–27* were from patients defined *sub-fertile* with at least one of the principal basal seminal parameters compromised (number of spermatozoa <15 × 10^6^/mL or *PR* < 32%). In the present study, physiological morphology was not considered a parameter for discriminating between the two groups.

### 2.2. Routine Sperm Analysis

#### 2.2.1. Macroscopic Analysis

Samples were incubated at 37 °C until the analysis was performed. The analysis to assess volume, pH, fluidification, and viscosity was started within one hour from semen collection.

#### 2.2.2. Determination of Sperm Count and Motility

Each semen sample was assessed for sperm motility and kinematics of movement using a disposable counting chamber (Counting Chamber Makler, Sefi Medical Instruments, Israel). Sperm count was performed on undiluted specimens. The grid was on a cover glass. The number of spermatozoa counted in any strip of 10 squares of the grid indicated their concentration in millions/mL. No additional factors were necessary for the calculation. We counted at least 3 strips and the mean value was used. The chamber was 10 microns deep, which eliminates blurring and allows sperm to move freely. The applied sample was observed in one focal plane. The motility of each spermatozoon was graded as follows: PR, active motility; NP, all other patterns of motility with no progression; immotility (no movement) [33].

#### 2.2.3. Determination of Sperm Morphology

To determine sperm morphology, each sample was analyzed by using Diff-Quik-stained slides (Test Simplets, Origio, Denmark). Restricted criteria by Kruger as indicated by the WHO manual were used to analyze at least 200 spermatozoa per sample [33]. 

#### 2.2.4. Determination of Sperm Viability 

Samples were assessed for sperm viability by staining with 1% Eosin-Y in saline (VitalScreen, FertiPro N.V., Belgium). Briefly, 50 μL semen samples were mixed with 2 drops of 1% Eosin-Y in a sterile test tube and a drop of semen-stain mixture was placed on a microscope slide. The smear was covered with a cover glass before drying and was immediately analyzed under the microscope. At least 200 spermatozoa were counted and classified as stained (dead) or unstained (viable).

### 2.3. HPV-DNA Detection and Typing

DNA extraction was performed on sperm samples (100–300 µL) using an automatic instrument (Maxwell MDX16, Promega Italia srl, Milan, Italy) based on paramagnetic particles. 10 µL of the solution were used for PCR amplification of HPV sequences from the L1 region using SPF10 primers in a final reaction volume of 50 µL for 40 cycles.

Positive and negative controls were introduced in each set of 12 reactions, including DNA from Siha and HeLa cell lines at a specified number of HPV copies, and blank reagents throughout all steps of the procedure.

Concurrent amplification of human HLA-DPB1 gene was included in the assay as internal control for DNA adequacy.

HPV type-specific sequences were detected by the line probe, INNO-LiPA HPV genotyping CE assay, version INNOLIPA HPV GENOTYPING EXTRA II (Fujirebio Italia S.r.l., Italy), according to the manufacturer’s instructions. The EXTRA version of the assay allows the simultaneous and separate detection of 32 HPV types: 13 high-risk HPV types (HR; 16, 18, 31, 33, 35, 39, 45, 51, 52, 56, 58, 59 and 68), 6 intermediate-risk HPV types (IR; 26, 53, 66, 70, 73 and 82), of 9 low-risk HPV types (LR; 6, 11, 40, 42, 43, 44, 54, 61 and 81), and 4 unclassified HPV types (62, 67, 83, and 89). Hybridization patterns were automatically analyzed by the LiRAS system and checked by two independent readers.

### 2.4. Water Permeability Measurements

Osmotic water permeability of human sperm samples was measured by a stopped-flow light-scattering method as previously described [30,34]. The initial rate constant of sperm cells volume changes (K) was obtained by fitting the time course of light-scattering with a one phase exponential decay (GraphPad Prism 4.00, 2003). The water permeability coefficient, Pf, was calculated as previously described by Wiener et al. [35], from the following equation:

Pf = k · V_0_/ΔC · V_W_ · A

where ΔC is the osmotic gradient, V_w_ the molar water volume, V_0_ the cell volume and A the cell surface area. Vo and A were obtained by Curry et al. [36].

Water transport was evaluated in sperm cells from normospermic and sub-fertile subjects, with or without HPV infection, following exposure to hypoosmotic medium (150 mOsm osmotic gradient).

### 2.5. Immunofluorescence

Immunolocalization of AQP3, 7, 8, and HPV was evaluated in human sperm samples, which were smeared on polylysine-coated slides, air dried and fixed in 4% paraformaldehyde in PBS for 30 min, and then washed with PBS. Antigen retrieval was performed by placing the slides in a Coplin jar containing retrieval buffer (0.05% tween-20, 10 mM citrate-HCl buffer, pH 6.0) and microwaving at high power level. When the solution started to boil (5 min) the oven was turned off. Two more cycles were repeated, and the jar was left at room temperature allowing slides to cool for at least 20 min. After washing with PBS, the slides were then blocked with 3% BSA in PBS at room temperature for 30 min. Double labeling experiments were performed by incubating the slides overnight (in the cold) with affinity pure anti-AQP3 or AQP7 or AQP8 primary antibodies (Anti-AQP3, SAB5200111, Sigma-Aldrich, USA; Anti-AQP7, ab191063, Abcam, UK; Anti-AQP8, ab133667, Abcam, UK) and with a prediluted mouse monoclonal anti-HPV antibody ([K1H8] ab75574; Abcam, UK) that recognizes the major capsid L1 protein of the following HPV types: 6, 11, 16, 18, 31, 33, 42, 51, 52, 56, 58. 

In some experiments, other Anti-AQP8 and anti-HPV were used: Anti-AQP8 rabbit polyclonal IgG affinity pure (AQP8-A, 1:500; Alpha Diagnostics Intl. Inc., San Antonio, TX, USA) and Anti-HPV Type 16 L1 Monoclonal Antibody (CAMVIR-1; catalog # MA1-34821; Invitrogen, Life Technologies, Italia). Anti-AQP antibodies were used at the following dilutions in antibody diluent (DakoCytomation, Milan, Italy): AQP3, 1:300; AQP7 1:250; AQP8 1:250. After three 5 min washes with PBS, slides were incubated at room temperature with the fluorescent secondary antibody (ab150117, 1:400, Abcam, Cambridge, UK) and rhodamine red –X-conjugated affinity pure goat anti-rabbit IgG (H+L) (1:50 dilution; 111-295-045; Jackson ImmunoResearch Europe Ltd., Cambridgeshire,, UK) for 30 min.

Slides were then washed 3 × 5 min with PBS, mounted in ProLong^®^ Gold antifade reagent with 4′,6-Diamidino-2-Phenylindole (DAPI; Molecular Probes) and examined with a TCS SP5 II confocal microscopy system (Leica Microsystems) equipped with a DM IRBE inverted microscope (Leica Microsystems). Images were visualized and analyzed by LAS AF software (Leica Microsystems Application Suite Advanced Fluorescence). Control experiments were performed simultaneously using non-immune serum. 

### 2.6. Co-Immunoprecipitation Assay and Immunoblotting

Co-immunoprecipitation (Co-IP) of AQP8 and HPV L1 protein was performed using Capturem IP & Co-IP Kit (Cat. No. 635721; Takara Bio Europe), according to the manufacturer’s instructions. Briefly, sperm cells were pelleted, resuspended in 400 µL of lysis buffer and 4 µL of protease inhibitors, and left on ice for 15 min. Cells were centrifuged at 17,000× *g* for 10 min at 4 °C and the supernatant (OS) was removed and kept. Protein content in the supernatant was measured as described below and 600 µg of protein lysate was incubated with 30 µL of anti- HPV Type 16 L1 Monoclonal Antibody (CAMVIR-1) for 20 min at room temperature (r.t.) with end-over-end rotation. The equilibrated spin column was loaded with the lysate and centrifuged at 1000× *g* for 1 min at r.t. The sample flow-through (FT) was collected and 100 µL of wash buffer added to the column (the solution was kept; W). After centrifuging at 1000× *g* for a minute at r.t., 6 µL of Tris 1 M pH 8.0 were added to the collection tube and 60 μL Elution Buffer was added to the column and centrifuged again. Finally, the eluted solution (E) was collected for immunoblotting analysis.

Immunoblotting was carried out as previously described [30]. Briefly, samples were heated at 80 °C in Leammli buffer [37] and loaded (30 µg OS, FT and 30 µL W and E) on Mini-PROTEAN TGX/TGX Stain-Free precast Gels 4-20 (Cat. #456-8094, Bio-Rad). Gels were transferred onto PVDF membrane using Trans-Blot Turbo (Bio-Rad Laboratories S.r.l., Italy). Blots were blocked for 1 h with a solution containing 5% skimmed dry milk, 0.1% Tween in TBS and then incubated overnight with the recombinant Anti-AQP8 antibody (ab133667, Abcam) or the anti-HPV Type 16 L1 Monoclonal Antibody (CAMVIR-1; Invitrogen), diluted 1:750 and 1:300, respectively. The membranes were washed thrice with 0.1% tween-20 in TBS and incubated for 1 h with peroxidase-conjugated goat anti-rabbit or rabbit anti-mouse IgG diluted 1:100000 in 0.1% tween-20 in TBS. After washing, the bands were detected using the Westar Supernova (Cyanagen s.r.l. Italy) and the images acquired using iBright Western Blot Imaging Systems (Invitrogen).

### 2.7. Protein Content

Protein content was determined with the Bradford method [38] with bovine serum albumin as a standard.

### 2.8. Protein Modelling and Docking Simulations

AQP8 was modelled using the I-Tasser server [39] and the tetramer was generated by Z-Dock [40]. The resulting model was then used to test interaction modes of AQP8 with the L1 pentamer (PDB ID: 1DZL) by the ROSIE server [41]. The resulting one thousand interaction models were manually evaluated; a lipid-embedded AQP8 model, generated by the CHARMM-GUI interface (http://www.charmm-gui.org/) was used as a reference to filter out the 987 interaction modes clashing with the lipid bilayer. The remaining 17 poses were ranked according to ROSIE total energy score. The best scoring model was visualized by Pymol (The PyMOL Molecular Graphics System, Version 2.0 Schrödinger, LLC) and analyzed by PISA [42].

### 2.9. Statistics

All data were expressed as mean ± S.E.M. or S.D. (Table of semen parameters). The significance of the differences of the means was evaluated by using one-way ANOVA followed by Newman–Keuls’s *Q* test or Student’s *t* test. To evaluate the significance of the differences of the means of the semen morphology parameters and of the osmotic water permeability, the Kruskal–Wallis test followed by Dunn’s multiple comparison test was used. All statistical tests were carried out using GraphPad Prism 4.00, 2003 (GraphPad Software, La Jolla, CA, USA, www.graphpad.com).

## 3. Results

### 3.1. Semen Characteristics in Normospermic and Sub-Fertile Subjects with and without HPV Infection

The semen characteristics of all subjects included in the study, either normospermic (n = 67) or sub-fertile (n = 27), who attended the clinic because of infertility, are presented in Table 1. Sperm concentration, progressive and total motility, and motile sperm count were all dramatically reduced in sub-fertile patients compared with normospermic subjects as previously shown [30]. No differences in the parameters were observed in the sperm from either normospermic or sub-fertile, non-infected, and HPV-infected subjects. 

### 3.2. HPV-DNA Detection and Typing

HPV typing of human sperm samples was done by SFP10-LIPA, a robust and sensitive method for the simultaneous detection of several HPV types. Results showed that 47 out of 94 sperm samples were HPV-infected: it was possible to identify the type in only 36 samples, whereas in 11 samples the types were unidentifiable by the test. Multiple HPV types were detected in 7 samples. Twenty-five samples were infected with HR, 6 with IR, 5 with LR and 2 with unclassified HPV types. The most common HR type was HPV 16 (n = 17) followed by HPV 31 and HPV 52 (n = 3), HPV 18 (n = 2) and HPV45, 51, 56 (n.1). Three IR HPV types were found: HPV 53 (n = 3), HPV 73 (n = 2) and HPV 66 (n = 1). The most common LR type was HPV 6 (n = 4) followed by HPV 44 and 81 (n = 1). HPV 67 and 89 (n = 1) were found as unclassified HPV types. 

### 3.3. Osmotic Water Permeability of Human Ejaculated Semen from Normospermic and Sub-Fertile Subjects with and without HPV Infection

**Figure 1:**
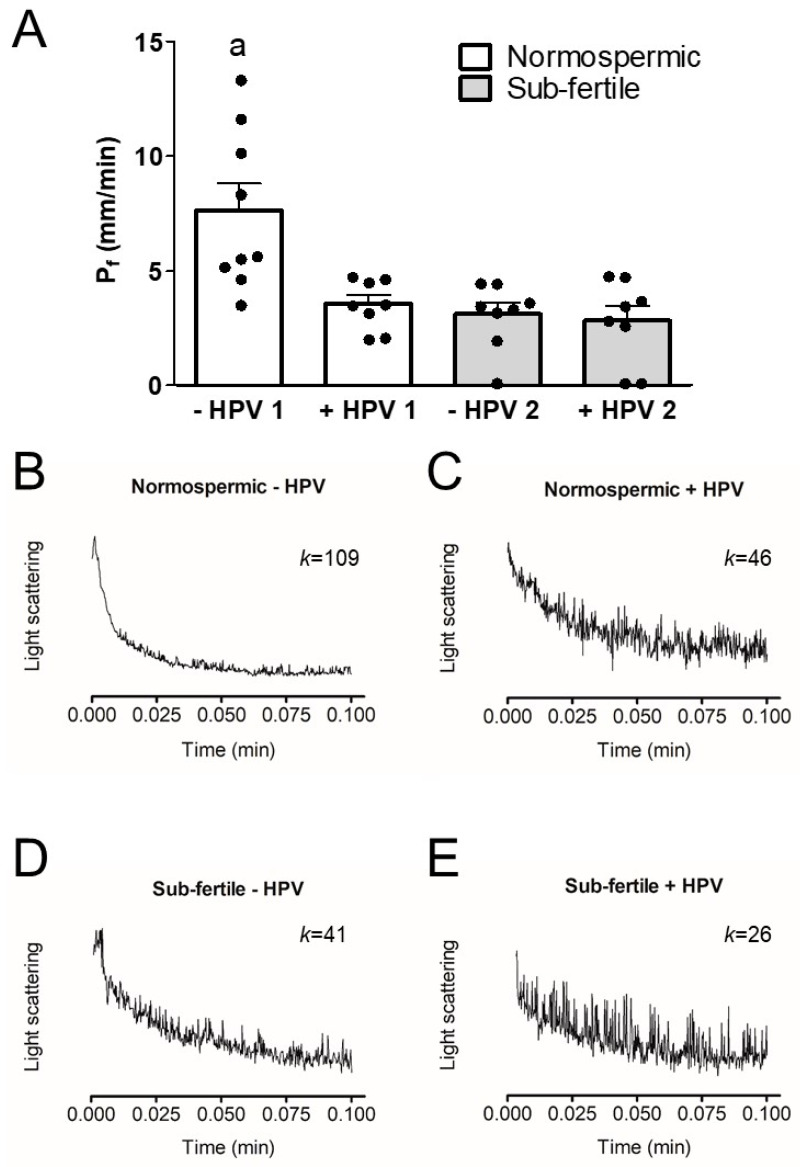
Osmotic water permeability of human ejaculated semen from normospermic (1) and sub-fertile (2) subjects with (+HPV) and without (−HPV) HPV infection. The osmotic water permeability was measured by exposing sperm cells to a 150 mOsm osmotic gradient and expressed as Pf (see Materials and methods). (**A**) Bars represent means ± SEM of 4–15 single shots for each of 8–10 different experiments; the individual data points are also shown (bullet point). a, *p* < 0.05 vs. normospermic HPV-positive, sub-fertile HPV-negative and sub-fertile HPV-positive (Kruskal–Wallis followed by Dunn’s Multiple Comparison Test). (**B**–**E**) Representative traces of stopped-flow osmotic water permeability measurements obtained from ejaculated semen of normospermic (**B**,**C**) and sub-fertile (**D**,**E**) subjects with (**C**,**E**) and without (**B**,**D**) HPV. K relative values of single curves are also shown.

Sperm cells act as functional osmometers displaying a rapid swelling when exposed to a hypotonic medium (Figure 1B–E). The resulting scattered light intensity decay could be fitted to a single exponential decay function to calculate the initial rate constant K and then the Pf as indicated in Materials and Methods.

To test whether HPV infection was able to inhibit AQPs function, we measured the osmotic permeability of human sperm cells from both normospermic and sub-fertile subjects with and without HPV infection. Figure 1A shows that in subjects without HPV infection, the osmotic permeability of sperm cells from the sub-fertile group was lower than that of the normospermics, as previously demonstrated (Laforenza et al. 2016). Interestingly, the osmotic permeability of sperm cells from the HPV-infected subjects both normospermic and sub-fertile was reduced by 53% and 63% respectively compared to that of non-infected normospermic subjects. Given that the normospermic HPV-positive subjects had a reduced sperm osmotic permeability despite semen parameters in the normal range, we hypothesized that HPV infection could damage semen parameters over time. To test this hypothesis, we compared the parameters of the subjects who were re-evaluated after more than 4 months with their initial parameters: there was a significant reduction only in sperm number (69.34 ± 13.1 vs. 49.2 ± 8.5; *p* = 0.0171, Student’s t test for paired data).

### 3.4. Immunolocalization of AQP3, 7, 8, and HPV Evaluated in Human Ejaculated HPV-Infected Semen

**Figure 2:**
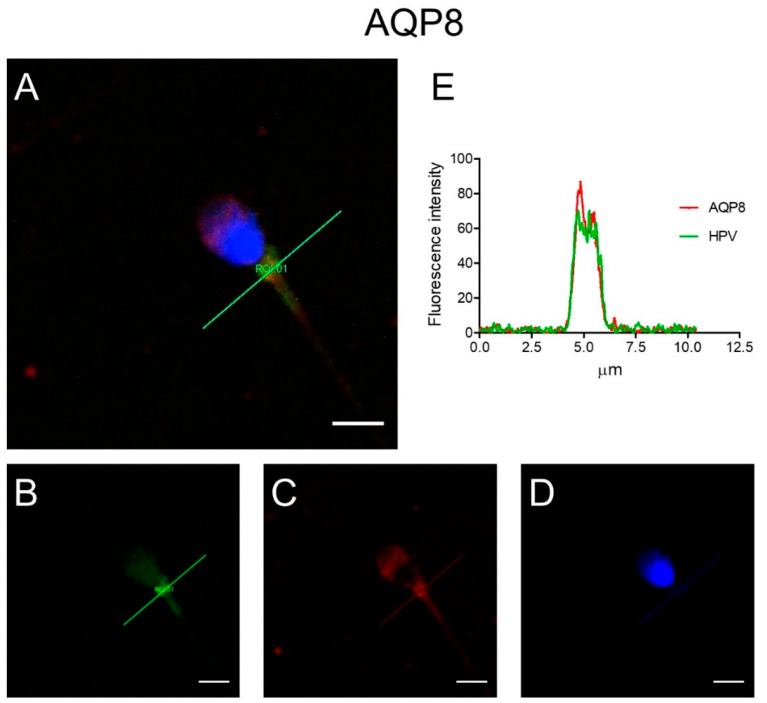
Representative immunofluorescence confocal microscopy images of colocalization of AQP8 and HPV in human sperm. (**A**) Green labeling indicates the presence of HPV, red labeling the expression of AQP8, while nuclei were counterstained by DAPI (blue). Yellow labeling shows colocalization signal of AQP8 with HPV. Scale bar, 5 µm. (**B**–**D**) Images show single labeling for HPV (green; **B**), AQP8 (red; **C**) and nuclei (DAPI; **D**). (**E**) Colocalization graphs, measured in the green line position in panel A, showing the overlap of the fluorescence signals originated by AQP8 and HPV staining.

HPV localization was determined to verify its possible interaction with AQP3, AQP7, and AQP8. To this end, colocalization experiments were performed. Double label immunofluorescence showed that HPV and AQP8 proteins colocalized in the midpiece of the spermatozoa, as per the resulting yellow fluorescence in merged images (Figure 2A) of HPV (green; Figure 2B), AQP8 (red; Figure 2C) and Nuclei stained with DAPI (Figure 2D). Colocalization graphs showed the overlap of the fluorescence signals originated by HPV (green) and AQP8 (red) labeling (Figure 2E).

**Figure 3:**
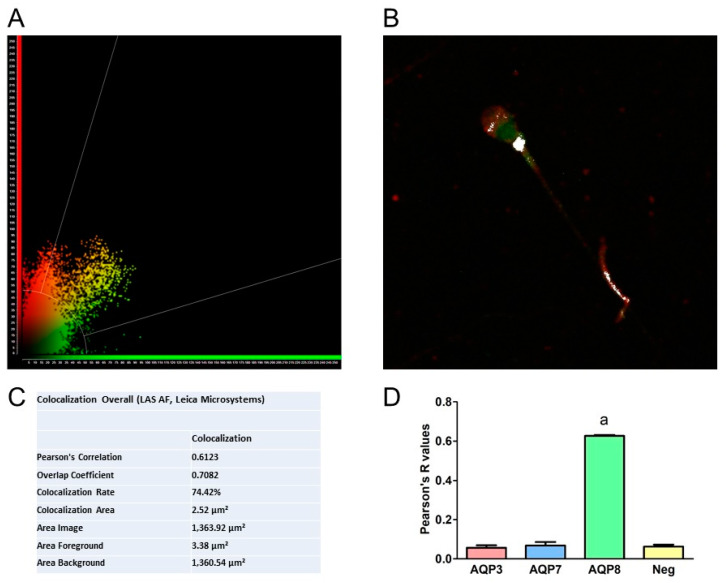
Representative colocalization analysis using the Leica LAS-AF image system. (**A**) Pixels distribution scatter plot: colocalized yellow pixels between the two diagonals, red (AQP8) pixels in ordinate and green (HPV) pixels in abscissa. (**B**) Leica LAS-AF software generates an image of colocalized pixels, highlighted in white, superimposed on an RGB-merge of two channels. (**C**) Overall colocalization values obtained from the representative colocalization analysis using the Leica LAS-AF image system. (**D**) Statistical analysis of Pearson’s coefficient R values was obtained from at least 6 different double immunofluorescence experiments with anti-HPV antibody and anti-AQP3, AQP7, or AQP8 antibodies. Negative controls (Neg) were also analyzed. a, *p* < 0.0001 vs. AQP3, AQP7 and Neg (ANOVA, followed by Newman–Keuls’s Q test).

Colocalization of AQP8 and L1 proteins was quantified using Pearson’s correlation coefficients and intensity profile plots by LAS AF software (Leica Microsystems Application Suite Advanced Fluorescence). Image analysis revealed a Pearson’s correlation coefficient of 0.612 (Figure 3). Figure 3 shows a representative colocalization analysis with the distribution scatter plot (A), the image with colocalized pixels highlighted in white (B) and the overall colocalization values obtained from the representative analysis (C). Finally, Pearson’s coefficient R values obtained from colocalization experiments of L1 protein with AQP8 showed a statistically significant difference with those obtained with AQP3 and AQP7 (Figure 3D).

**Figure 4:**
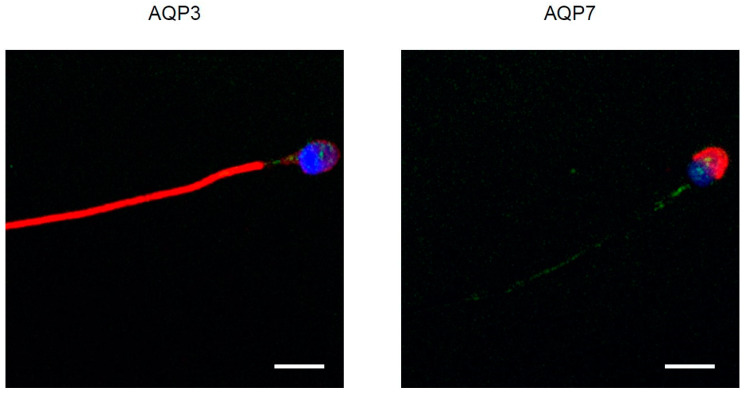
Representative immunofluorescence confocal microscopy images of AQP3, AQP7, and HPV in human sperm. Green labeling indicates the presence of HPV, red labeling the expression of AQP3 or AQP7, while nuclei were counterstained by DAPI (blue). Merged pictures are shown. No colocalization (no yellow signal) was observed for either AQP3 or 7 and HPV. Scale bar, 5 µm.

In contrast, AQP3 and AQP7 staining did not show any overlap of the fluorescence signal with HPV, as per the absence of yellow fluorescence in merged images of AQP3 and 7 (red) and HPV (green) (Figure 4 and Figure S1). In some experiments, a different antibody that specifically detects the L1 protein of HPV 16 (see Methods) was used. The results confirmed a colocalization of L1 with AQP8, but not with AQP3 and AQ7. Control experiments were performed simultaneously using non-immune serum which did not show any labeling (Figure S2). 

### 3.5. Co-Immunoprecipitation of AQP8 and L1 Protein 

**Figure 5:**
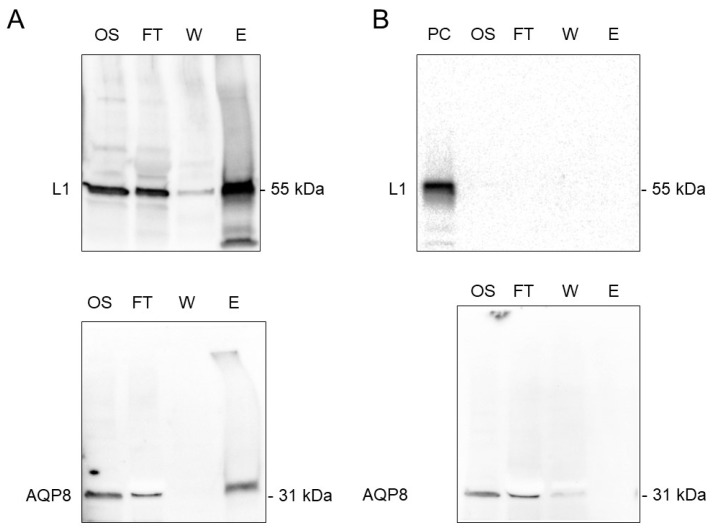
Co-immunoprecipitation of AQP8 and HPV L1 proteins in human sperm cells. (**A**) The sperm cell lysates of HPV16 infected subjects were subjected to co-immunoprecipitation with a HPV L1 specific antibody; protein expression of both AQP8 and L1 was detected by immunoblotting with anti-AQP8 and with anti-HPV antibodies as indicated in Materials and methods. (**B**) As negative control, HPV-negative sperm cells lysates were incubated with the anti-HPV antibody. Major bands of AQP8 and L1 proteins were shown. OS, original sample; FT, flow-through; W, wash; E, elution, PC, positive control.

To confirm the AQP8 and L1 protein interaction, we performed co-immunoprecipitation with anti-L1 protein antibody followed by western blot. L1 protein pull-down resulted in the co-immunoprecipitation of AQP8 (Figure 5A). Bands of the predicted molecular size were observed for both AQP and L1 protein in the original sample, in the FT and in the elution. No immunoreactive bands in the elution were observed when co-immunoprecipitation was performed in HPV-negative sperm cells lysates (Figure 5B) or without anti-L1 protein antibody (Figure S3).

### 3.6. Protein Modelling and Docking Simulations

**Figure 6:**
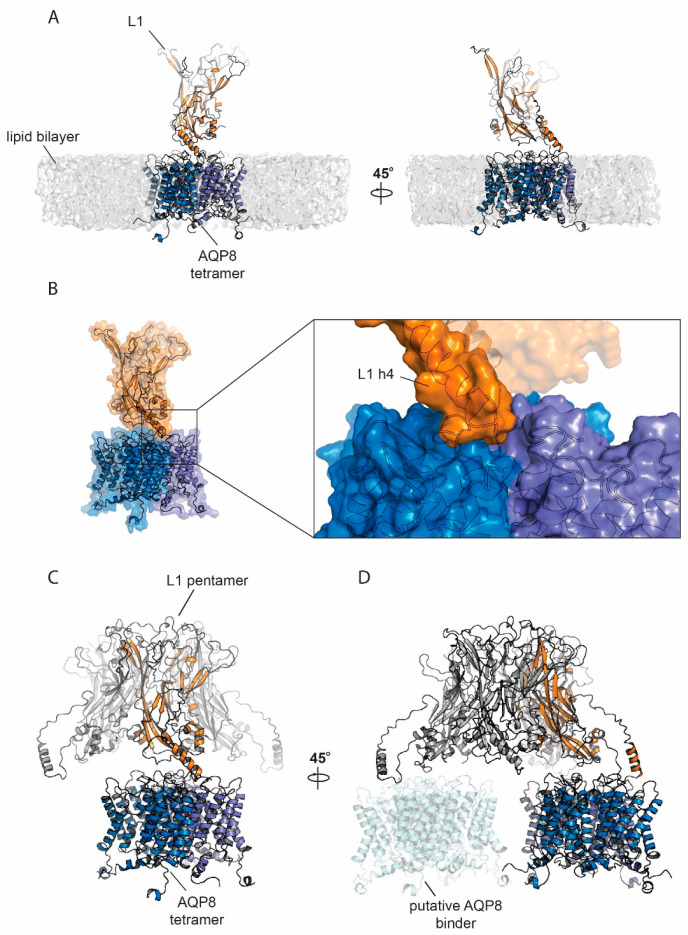
Cartoon representation of AQP8 model and L1 interaction. (**A**) AQP8 model (each monomer is colored in a different shade of blue) and L1 (orange) interaction represented in the presence of a lipid bilayer. (**B**) Surface representation of L1:AQP8 complex. In the box, the magnification of the interaction surface where one AQP8 monomer interacts with the L1 extended h4 helix domain. (**C**) Pentameric L1 used for docking simulations. (**D**) Possible interaction of the L1 pentamer with multiple AQP8 tetramers.

Given the experimental evidence of colocalization between AQP8 and HPV L1, we tested a potential molecular interaction mode by molecular modelling and docking. A model for AQP8 was generated by I-Tasser; the top scoring model (C-score 0.85) was used as a base for tetramer generation by Z-Dock. The tetramer assembly with the highest similarity to available AQP structures was used for further analysis. Pentameric L1 docking onto AQP8 by ROSIE was followed by manual selection of the 1000 generated models versus a lipid-embedded AQ8 tetramer to exclude docking poses clashing with the lipid membrane. Seventeen models passed this selection criterium. The top-ranking pose (total energy score: –1045.287) showed an interaction mode where the L1 pentamer is located on top of the AQP8 tetramer (Figure 6C). The interaction, however, occurs through a single L1 monomer and it especially involves the L1 extended lateral domain h4 [43] (Figure 6B). Notably, the L1 h4 helix is the point of contact between different pentamers in the HPV capsid [43]. Such interaction is hydrophobic and involves a long loop. It has been proposed that the L1 h4 is a highly flexible adjustable unit [43], which therefore might adapt its conformation to bind multiple AQP8 tetramers (Figure 6D). In AQP8, the interacting surface spans two adjacent monomers (Figure 6A,B).

## 4. Discussion

Several AQPs have been found in human sperm and seem to be involved in volume regulation and, as recently reported, also in ROS elimination [30]. In addition, sperm number and motility are related with AQP functioning, although, unfortunately, a close association with a single AQP paralog could not be demonstrated [30]. To date, AQP3, AQP5, AQP8 and AQP9 have been proven to possess H_2_O_2_ transport capacity in addition to that of water (peroxiporins) [22,23,24,25,26,27,28,29]. Moreover, two of them are expressed in sperm cells: AQP3 is localized both in the sperm tail membrane and, as a few granules, in the head and midpiece; AQP8 is localized in the midpiece (in the mitochondria and/or the plasma membrane) of spermatozoa [30,31].

Some studies suggest that HPV infection may have a deleterious effect on sperm quality (and function) possibly leading to male infertility [5,7,8,9,10,11,12,13,14,15,16,17,44]. To date, the underlying mechanisms are almost unknown. HR-HPV proteins elicit inflammation and an increase in ROS concentration inside the host cells, leading to oxidative stress [4]. In this context, there is no doubt that AQP-mediated H_2_O_2_ efflux to the extracellular fluid can be considered a fundamental ROS removal system, especially in an oxidative stress status [21,22].

To address the possible damaging effect of HPV infection in AQP-mediated H_2_O_2_ removal, we measured osmotic permeability to water, which is an index to that of H_2_O_2_ [24,30]. In fact, HPV infection was found to reduce osmotic permeability in the sperm of normospermic subjects (Figure 1). This result may suggest that: (1) HPV infection influences AQP functioning making the sperm more sensitive to oxidative stress even in normospermic subjects, (2) AQP inhibition by HPV could negatively affect functional parameters over time, leading to sub-fertility. A future longitudinal study of sperm parameters variation in infected subjects could clarify this issue.

The second purpose of this study was to elucidate the interaction between AQPs and HPV by confocal fluorescence microscope and by 3D structural simulation analysis. The results of double label immunofluorescence showed that L1, the major capsid protein of HPV, and AQP8 were highly colocalized in sperm (Figure 2). Apparently, HPV interacts with AQP8, but not with AQP3 and AQP7. This suggests that the inhibitory effect of HPV infection on AQP-mediated water permeability might be due to a direct interaction of HPV with the AQP8 pore. At present, with no conclusive data on the subcellular localization of AQP8, it is not possible to support the tentative hypothesis that HPV inhibition reduces the elimination of H_2_O_2_ from the sperm cells or their mitochondria. Further investigation and more experiments will be needed to prove this hypothesis and reach a sound conclusion. Recently, in a study performed in the oviparous marine teleost, *Sparus aurata*, the effect of oxidative stress on the functionality of Aqp8b, an orthologue of human AQP8, was examined [45]. Gilthead seabream sperm cells exposed to seawater were subjected to hyperosmotic stress and to a damaging ROS load. In these conditions, Aqp8b underwent a phosphorylation that caused its relocation to the inner mitochondrial membrane. This localization is strategically fundamental in guaranteeing H_2_O_2_ efflux thus maintaining flagellar motility. Inhibition of Aqp8b led to an insufficient elimination of ROS and to a reduction of ATP synthesis which in turn caused a reduction of sperm motility until its progressive arrest. With all the precautions given that these results were obtained on fish sperms, we could speculate that in humans the inhibition of AQP-mediated H_2_O_2_ efflux by HPV could impair sperm function and/or cell number. In fact, according to our molecular modelling and docking simulation results, the L1 protein seems to have a favorite site of interaction in the region located midway of two adjacent monomers of AQP8, with the potential, in its pentameric form, to bind to multiple pore copies. Depending on AQP8 localization, we might speculate that during the infection phase, the whole virus could interact with the AQP8 located on the external surface of the plasma membrane and, during intracellular replication, monomeric or pentameric L1 produced ex novo could form complexes with intracellular (mitochondrial) AQP8.

AQP3, AQP7, and AQP8 null mice studies show a normal sperm production and a normal motility in response to physiological hypotonicity except for an increased vulnerability to hypotonic swelling occurring after entering the uterus observed in AQP3 deficient mice [31,46,47,48].

The relevance of the peroxiporins (AQP3 and AQP8) in sperm cell functioning of AQP-null mice appears limited. Human male has a relatively low fertility and therefore may be more susceptible to toxic agents (in this case HPV infection and the resulting oxidative stress) for reproduction than male rodents [49]. In this context, AQPs can represent only one of the numerous targets damaged by HPV but with a key role in worsening the oxidative stress condition.

## 5. Conclusions

HPV infection of sperms affects both AQPs expression and functionality. Alterations in water and hydrogen peroxide diffusion capacity are related to a direct interaction of viral L1 protein with AQP8. Thus, our data suggest that HPV reduces AQP8-mediated detoxification mechanism leading to sperm distress and sperm dysfunction.

## Figures and Tables

**Table 1:** Semen parameters of normospermic and sub-fertile patients with (+ HPV) and without HPV (−HPV) infection.

Semen Parameters	Normospermic HPV− (n = 35)	Normospermic HPV+ (n = 32)	Sub-Fertile HPV− (n = 12)	Sub-Fertile HPV+ (n = 15)
Semen volume (mL)	4.35 ± 1.69	3.69 ± 1.18	4.07 ± 0. 99	4.21 ± 1.98
Sperm concentration (mil/mL)	71.78 ± 50.50	80.61 ± 51.47	23.01 ^a^ ± 24.30	21.84 ^a^ ± 23.94
Progressive motility (PR%)	55.54 ± 13.02	54.41 ± 13.13	29.58 ^a^ ± 11.31	25.47 ^a^ ± 12.89
Motile sperm count (mil/mL)	42.85 ± 35.93	48.86 ± 34.05	5.94 ^a^ ± 6.62	5.86 ^a^ ± 6.47
Non-progressive motility (NP%)	8.69 ± 5.32	7.19 ± 3.51	9.33 ± 3.47	11.40 ^b^ ± 6.20
Total motility (PR% + NP%)	64.23 ± 11.43	61.59 ± 12.79	38.92 ^a^ ± 12.33	36.87 ^a^ ± 13.79
Morphology (% normal)	2.37 ± 1.77	2.70 ± 1.86	1.33 ^c^ ± 2.15	0.80 ^d^ ± 1.21

Values are mean ± S.D. ^a^ *p* < 0.01–0.001, vs. Normospermic HPV− and Normospermic HPV+ ^b^ *p* < 0.05–0.01 vs. Normospermic HPV+. (One-way ANOVA, Newman–Keuls post test). ^c^ *p* = 0.05–0.01 vs. Normospermic HPV+. ^d^ *p* = 0.01–0.001 vs. Normospermic HPV− and Normospermic HPV+ (Kruskal–Wallis test followed by Dunn’s multiple comparison test).

## Supplementary Materials

The following are available online at https://www.mdpi.com/2073-4409/9/5/1241/s1. Figure S1: Immunofluorescence confocal microscopical images of AQP3, AQP7, and HPV in human sperm, Figure S2: Immunofluorescence negative control, Figure S3: Negative control of co-immunoprecipitation of AQP8 and HPV L1 proteins in human sperm cells.

## Abbreviations

HR-HPV	High-risk Human Papillomavirus
AQP	aquaporin
ROS	reactive oxygen species
WHO	World Health Organization
PR	progressive
NP	non-progressive
