# Supplementary material for: HPV Infection Affects Human Sperm Functionality by Inhibition of Aquaporin-8
Source: Cells. 2020 May 17;9(5):1241. doi: 10.3390/cells9051241 (PMC7291193; doi:10.3390/cells9051241)
Supplement: Supplementary file 1 [file cells-09-01241-s001.pdf]

Supplementary Material

HPV infection affects human sperm functionality by inhibition of aquaporin-8

Giorgia Pellavio, Federica Todaro, Paola Alberizzi, Claudia Scotti, Giulia Gastaldi, Marco Lolicato, Claudia Omes, Laura Caliogna, Rossella E. Nappi and Umberto Laforenza

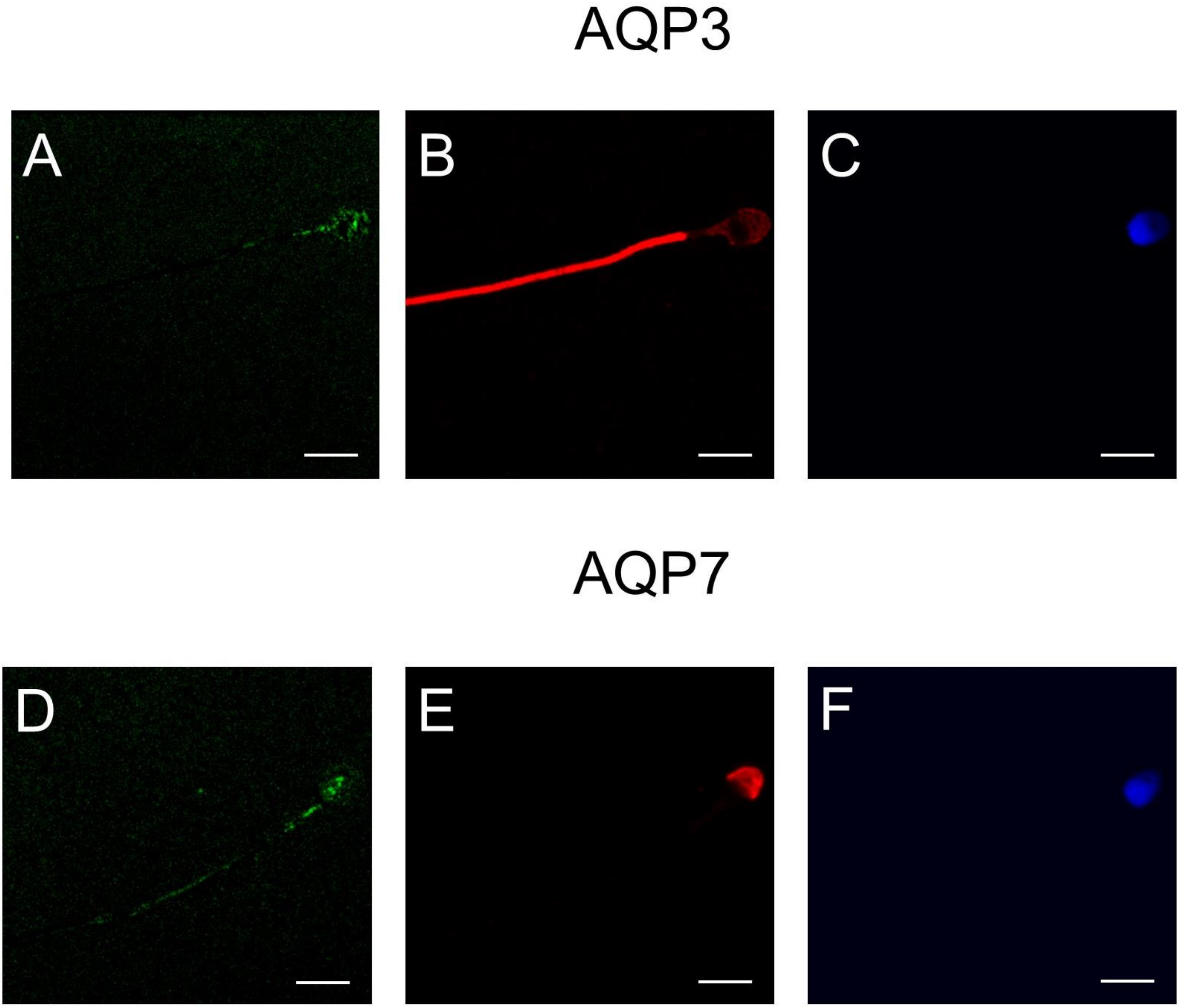

Figure S1: Immunofluorescence confocal microscopical images of AQP3, AQP7, and HPV in human sperm. Images show single labeling of the merged images of Figure 4. Green labeling indicates the presence of HPV (A, D), red labeling the expression of AQP3 (B) or AQP7 (E), while nuclei were counterstained by DAPI (blue; C, F). Scale bar, 5 µm.

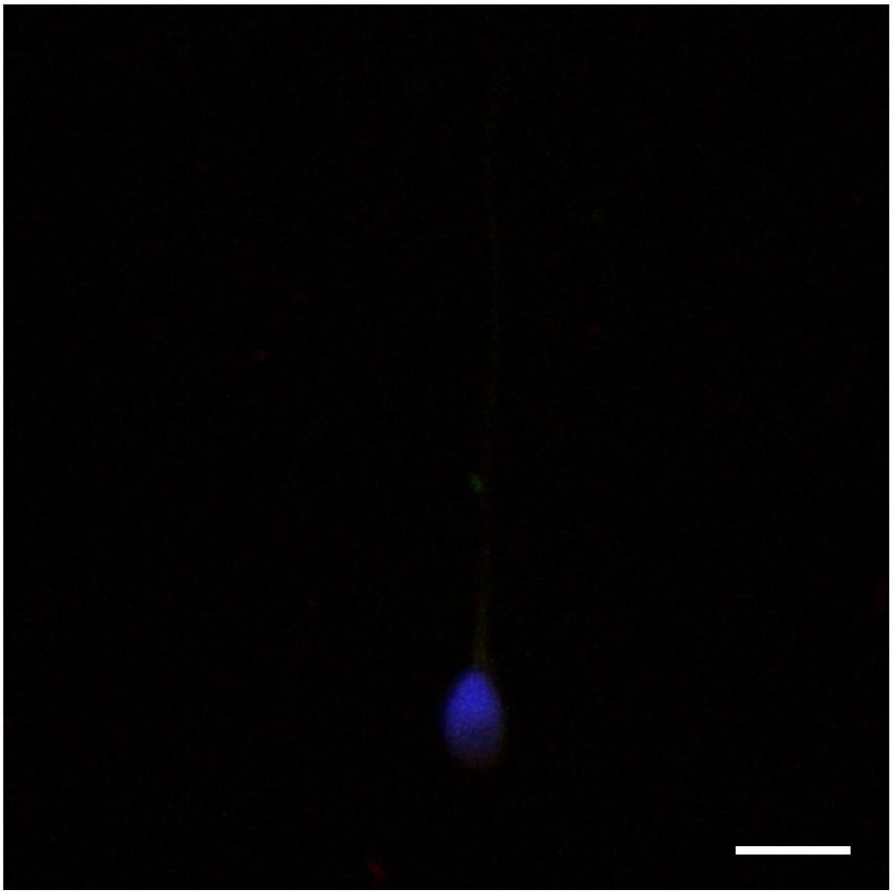

Figure S2: Immunofluorescence negative control. No or faint staining was observed when anti-aquaporins and anti-HPV antibodies were substituted with preimmune serum. Nuclei were counterstained by DAPI (blue). Scale bar, 10 µm.

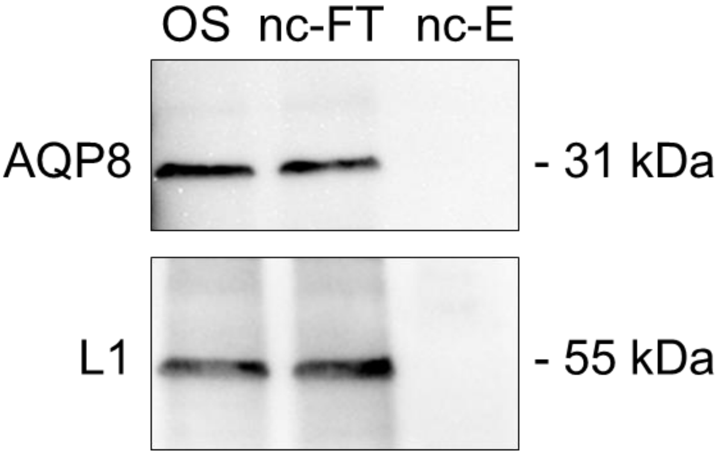

Figure S3: Negative control of co-immunoprecipitation of AQP8 and HPV L1 proteins in human sperm cells. As negative control (nc), lysates were incubated without the anti-HPV antibody. Major bands of AQP8 and L1 proteins were shown. OS, original sample; FT, flow-through; W, wash; E, elution.
